# Sociodemographic Disparities in Queue Jumping for Emergency Department Care

**DOI:** 10.1001/jamanetworkopen.2023.26338

**Published:** 2023-07-28

**Authors:** Rohit B. Sangal, Huifeng Su, Hazar Khidir, Vivek Parwani, Beth Liebhardt, Edieal J. Pinker, Lesley Meng, Arjun K. Venkatesh, Andrew Ulrich

**Affiliations:** 1 Department of Emergency Medicine, Yale University School of Medicine, New Haven, Connecticut; 2 Department of Operations, Yale University School of Management, New Haven, Connecticut; 3 Emergency Department, Yale New Haven Hospital, New Haven, Connecticut; 4 Center for Outcomes Research and Evaluation, Yale University, New Haven, Connecticut; 5 National Clinician Scholars Program, Yale University School of Medicine, New Haven, Connecticut

## Abstract

**Importance:**

Emergency department (ED) triage models are intended to queue patients for treatment. In the absence of higher acuity, patients of the same acuity should room in order of arrival.

**Objective:**

To characterize disparities in ED care access as unexplained queue jumps (UQJ), or instances in which acuity and first come, first served principles are violated.

**Design, Setting, and Participants:**

Retrospective, cross-sectional study between July 2017 and February 2020. Participants were all ED patient arrivals at 2 EDs within a large Northeast health system. Data were analyzed from July to September 2022.

**Exposure:**

UQJ was defined as a patient being placed in a treatment space ahead of a patient of higher acuity or of a same acuity patient who arrived earlier.

**Main Outcomes and Measures:**

Primary outcomes were odds of a UQJ and association with ED outcomes of hallway placement, leaving before treatment complete, escalation to higher level of care while awaiting inpatient bed placement, and 72-hour ED revisitation. Secondary analysis examined UQJs among high acuity ED arrivals. Regression models (zero-inflated Poisson and logistic regression) adjusted for patient demographics and ED operational variables at time of triage.

**Results:**

Of 314 763 included study visits, 170 391 (54.1%) were female, the mean (SD) age was 50.46 (20.5) years, 132 813 (42.2%) patients were non-Hispanic White, 106 401 (33.8%) were non-Hispanic Black, and 66 465 (21.1%) were Hispanic or Latino. Overall, 90 698 (28.8%) patients experienced a queue jump, and 78 127 (24.8%) and 44 551 (14.2%) patients were passed over by a patient of the same acuity or lower acuity, respectively. A total of 52 959 (16.8%) and 23 897 (7.6%) patients received care ahead of a patient of the same acuity or higher acuity, respectively. Patient demographics including Medicaid insurance (incident rate ratio [IRR], 1.11; 95% CI, 1.07-1.14), Black non-Hispanic race (IRR, 1.05; 95% CI, 1.03-1.07), Hispanic or Latino ethnicity (IRR, 1.05; 95% CI, 1.02-1.08), and Spanish as primary language (IRR, 1.06; 95% CI, 1.02-1.10) were independent social factors associated with being passed over. The odds of a patient receiving care ahead of others were lower for ED visits by Medicare insured (odds ratio [OR], 0.92; 95% CI, 0.88-0.96), Medicaid insured (OR, 0.81; 95% CI, 0.77-0.85), Black non-Hispanic (OR, 0.94; 95% CI, 0.91-0.97), and Hispanic or Latino ethnicity (OR, 0.87; 95% CI, 0.83-0.91). Patients who were passed over by someone of the same triage severity level had higher odds of hallway bed placement (OR, 1.01; 95% CI, 1.00-1.02) and leaving before disposition (OR, 1.02; 95% CI, 1.01-1.04).

**Conclusions and Relevance:**

In this cross-sectional study of ED patients in triage, there were consistent disparities among marginalized populations being more likely to experience a UQJ, hallway placement, and leaving without receiving treatment despite being assigned the same triage acuity as others. EDs should seek to standardize triage processes to mitigate conscious and unconscious biases that may be associated with timely access to emergency care.

## Key Points

**Question:**

Are historically marginalized patient populations more likely to be queue jumped (ie, receive care not based on acuity or first come, first-served principles) while waiting for emergency department (ED) care?

**Findings:**

In this cross-sectional study of 314 841 patients, 90 698 (28.8%) experienced a queue jump. Patients who were non-Hispanic Black, Hispanic or Latino, Spanish speaking, or insured by Medicaid were more likely to be jumped over; patients who were jumped over had higher odds of hallway bed placement and leaving before treatment is complete.

**Meaning:**

These findings suggest EDs should seek to standardize triage processes to mitigate conscious and unconscious biases that may be associated with patient access to emergency care.

## Teaser

This cross-sectional study evaluates the association between sociodemographic disparities and receiving emergency department care not in order of acuity or arrival time.

## Introduction

Patient triage is the ubiquitous first step of emergency care and is essential to determine emergency department (ED) treatment order. Although new patient intake models are being implemented to avoid triage, traditional triage continues to be common and necessary given the increasing supply and demand mismatch between clinical resources and ever-increasing patient arrivals in increasingly crowded EDs.^1,2^ Emergency severity index (ESI), a 5-level triage algorithm, is the most commonly used ED triage tool to prioritize ED treatment order based on anticipated resource needs. However, the tool is a subjective measure applied with wide variability which introduces opportunity for bias.^3^ For example, minoritized racial and ethnic groups who do not receive immediate care wait longer to be seen and Black patients are assigned lower triage scores and experience longer wait times, which can result in increased leaving without being seen (LWBS) rates.^4,5,6,7,8^ Furthermore, LWBS has been associated with ED revisit rates which is increasingly used as an ED and hospital performance measure.^9,10,11^ Such studies have shed light on triage disparities, but are limited with respect to patient outcomes and operational factors that may confound the analysis.

Queue formation and waiting periods in the ED are a regular occurrence given the persistent shift of acute unscheduled care toward the ED and increased ED crowding from high volumes of patients awaiting hospital bed placement.^1,2,12^ Although triage queues may delay access to emergency care, queue violations in which lower acuity or later arriving patients are seen before others may also be associated with reduced access to ED care. As such, although new front-end ED models such as physician-in-triage, split flow, and advanced analytic algorithms seek to minimize queue formation or triage variability and their association with patient care, they still result in waiting periods for ED treatment space according to a priority list.^13,14,15,16,17^ Similar priority queues are commonly used in other industries like call centers or grocery stores with increased waits associated with caller abandonment or reduced grocery purchases just as increased waits in the ED are associated with higher LWBS.^18,19,20,21^ However, to improve the quality and equity of emergency care delivery, we need to explore the degree to which queue prioritization or queue adherence may be associated with known disparities and whether these queue jumps are associated with waiting and common ED outcomes.^22^

We examined ED triage equity by characterizing the prevalence of queue jumps, care delays caused by queue jumps, and which patients receive earlier care despite being of lower acuity or arriving later. We specifically explored the association of patient social factors with queue jumping and outcomes including patient LWBS, hallway placement, 72-hour ED revisits, and escalation in care.

## Methods

We conducted a retrospective observational study of an EHR-based data set derived from the institutional data warehouse (Epic Systems). The study data set included all ED patient visits between July 2017 through February 2020 across 2 EDs within a large academic medical center with a total annual patient volume of more than 130 000 visits. We focused on this period before the COVID-19 pandemic to ensure relative consistency in expected arrival intensity and patient volumes. The study inclusion criteria are outlined in eFigure 1 in Supplement 1. ED registration processes encourage collection of self-reported race and ethnicity, although this may not always occur. Race and ethnicity data were collapsed into 1 combination race and ethnicity variable.^23^ This study follows the Strengthening the Reporting of Observational Studies in Epidemiology (STROBE) reporting guideline for cross-sectional studies and was approved by the institutional review board with a waiver of consent because data were deidentified.

In our study setting, ED patients typically followed a standard series of events at the initiation of ED care: (1) check-in, (2) triage, (3) wait in waiting area, and (4) movement to an ED treatment space. As part of the triage process, every patient is assigned an ESI acuity level ranking between 1 (the most severe, eg, cardiac arrest) and 5 (the least severe, eg, medication refill). Typically, the selection of patients from the waiting area followed a routing policy that we refer to as acuity-based first come, first served: the highest acuity patient who arrived earliest in the waiting area is next in line to be seen (eFigure 2 in Supplement 1). In practice, acuity-based first come, first served is not always followed. We observed such deviations in our data set; in this study we call these deviations unexplained queue jumps (UQJ). To illustrate how we classify these UQJs, consider an ED at time T where currently there are 6 patients (A, B, C, D, E, and F) who have all been triaged and assigned an ESI level (respectively 3, 4, 3, 4, 2, and 2). The arrival times to the ED of these 6 patients are given by: t_A _< t_B _< t_C _< t_D _< t_E _< t_F _< T. That is, patient A has arrived before B and B before C, and so forth. If a bed becomes available at time T, then according to acuity-based first come, first served, patient E will be given that bed and start receiving care ahead of all the others (eFigure 2 in Supplement 1). If, instead, patient C were to be given that bed, we would call the event a UQJ because patient C arrived after patient A (t_A _< t_C_) but is at the same acuity level, and patient C is of lower acuity than patients E and F. (eFigure 2 in Supplement 1).

According to the previously stated definition of a UQJ, each patient has the potential to experience a UQJ as 1 of 4 possible distinct event types: (a) being passed over by a lower acuity patient, (b) being passed over by a same-acuity patient who came later, (c) receiving care ahead of a same-acuity patient who came earlier, or (d) receiving care ahead of a higher-acuity patient. In theory a patient could experience any or all of the 4 previously stated events. Therefore, for each patient *i* we define count variables *UQJ_ia_* and *UQJ_ib_* for the total number of times the patient experiences each of the 2 ways of being passed over (a and b) and binary variables *UQJ_ic_* and *UQJ_id_* that are 1 if they ever receive care ahead of another patient in the 2 possible ways (c and d) or 0 if they never experience such an event. We also aggregated the same-acuity and lower-acuity subsets of UQJs to construct 1 combined measure for patients who are passed over and 1 aggregate measure for patients receiving care ahead of others.

In addition to the 4 types of UQJs, the ED outcome measures we analyzed include: (1) whether the patient was placed in a hallway ED bed instead of a room, (2) whether the patient left the ED before care completion (defined as leaving against medical advice, left without treatment complete [LWTC], or LWBS), (3) whether discharged patients returned to the ED for a revisit within 72 hours, and (4) whether admitted patients experienced an escalation in the care level requested during their time awaiting inpatient bed placement. Escalation in care level was defined as a change in admission order for a step-down unit or intensive care unit when the original admission order was for the general ward. This also included patients who went to the intensive care unit when the original bed order was for the step-down unit.

### Statistical Analysis

We constructed regression analyses to examine the association between patient social factors and the 4 UQJs. We also examined the association between UQJ exposure and ED outcomes. We estimated count regression models (zero-inflated Poisson) and binary outcome regression models (logistic regression) to identify the features that are associated with patients who experienced being passed over, and patients who were selected to receive care ahead of others, respectively. Zero-inflated Poisson model coefficients are expressed as incident rate ratios (IRR) and logistic regression model coefficients are expressed as odds ratios (OR). To adjust for the variability of ED operations, models included controls at the level of the ED encounter, ED staffing, ED occupancy, the mix of patients in the ED, and the time and date of the ED patient arrival. Models adjusted for patient variables including insurance status, race, ethnicity, sex, age, ESI, and language, which is consistent with the features used in prior literature.^24,25^ A comprehensive list of controls and their summary statistics are available in eTable 1, eTable 2, and eTable 3 in Supplement 1. All results were adjusted using the Benjamini-Hochberg procedure for multiple comparisons. Tests were 2-sided and significance was set at .05. Analyses were conducted from July to September, 2022, and were performed using R version 4.2.0 (R Project for Statistical Computing).^26^

We performed a sensitivity analysis using logistic regression to examine whether the primary analysis is consistent for high acuity arrivals. High acuity arrivals were defined as encounters that satisfied 1 of the following criteria: (1) the patient’s ESI level was assigned as 1, or (2) the patient’s ESI level was assigned as 2 and the patient was treated in the ED resuscitation room, or (3) their chief complaint was assigned as any of the following: trauma code activation, respiratory arrest, respiratory distress, cardiac arrest, stroke code activation, gunshot wound, or unresponsive. Additionally, at baseline we exclude the top and bottom 1st percentile (eFigure 1 in Supplement 1) since average effects are likely to be biased during extremely busy or slow times. As a sensitivity analysis, we repeated the analysis twice, first using all data, and second, excluding the top and bottom 0.5th percentile.

## Results

### Study Sample Description

The study population included 314 763 patient visits with a mean (SD) age of 50.46 (20.45) years, and 170 391 (54.1%) were female. A total of 184 862 patients (58.7%) arrived via walk-in. A total of 132 813 patients (42.2%) were non-Hispanic White, 106 401 (33.8%) patients (33.8%) were non-Hispanic Black, and 66 465 (21.1%) were Hispanic or Latino.

Among the patients included in our study, 167 332 (53.2% of all encounters) received care in an acuity-based first come, first served order. Overall, 90 698 patients (28.8% of all encounters) were passed over at least once by patients of same or lower acuity, and 71 490 patients (22.7% of all encounters) received care ahead of another patient of same or higher acuity. A total of 78 127 (24.8%) and 44 551 patients (14.2%) were passed over by a patient of the same acuity and lower acuity, respectively. A total of 52 959 (16.8%) and 23 897 (7.6%) patients received care ahead of a patient of the same acuity or higher acuity, respectively (Table).

**Table.** Summary Statistics of Data Set

Characteristic	Patients, No. (%)						
	Total	Patient passed over by same- or lower-acuity patient UQJ^a^	Patient passed over by lower acuity patient UQJ	Patient passed over by same-acuity patient UQJ	Patient receiving care ahead of same- or higher-acuity patient UQJ^a^	Patient receiving care ahead of higher-acuity patient UQJ	Patient receiving care ahead of same-acuity patient UQJ
Sample size	314 763 (100)	90 698 (28.8)	44 551 (14.2)	78 127 (24.8)	71 490 (22.7)	23 897 (7.6)	52 959 (16.8)
Age, mean (SD), y	50.46 (20.46)	46.95 (19.29)	47.31 (19.09)	45.97 (18.93)	52.14 (20.93)	44.73 (19.54)	55.74 (20.64)
Sex							
Female	170 391 (54.1)	51 517 (56.8)	25 554 (57.4)	44 885 (57.5)	39 360 (55.1)	13 044 (54.6)	29 300 (55.3)
Male	144 372 (45.9)	39 181 (43.2)	18 997 (42.6)	33 242 (42.5)	32 130 (44.9)	10 853 (45.4)	23 659 (44.7)
Race and ethnicity							
Hispanic or Latino	66 465 (21.1)	22 419 (24.7)	11 040 (24.8)	20 097 (25.7)	14 005 (19.6)	5743 (24)	9125 (17.2)
Non-Hispanic Black	106 401 (33.8)	32 132 (35.4)	15 578 (35)	27 835 (35.6)	22 088 (30.9)	9000 (37.7)	14 526 (27.4)
Non-Hispanic White	132 813 (42.2)	33 394 (36.8)	16 639 (37.3)	27 747 (35.5)	33 216 (46.5)	8361 (35)	27 747 (52.4)
Other^b^	9084 (2.9)	2753 (3.0)	1294 (2.9)	2448 (3.1)	2181 (3.1)	793 (3.3)	1561 (2.9)
Language preference							
English	283 258 (89.9)	80 346 (88.6)	39 432 (88.5)	68 902 (88.2)	64 618 (90.4)	21 443 (89.7)	48 122 (90.9)
Spanish	25 749 (8.2)	8617 (9.5)	4267 (9.6)	7706 (9.9)	5481 (7.7)	2018 (8.4)	3781 (7.1)
Other	5756 (1.8)	1735 (1.9)	852 (1.9)	1519 (1.9)	1391 (1.9)	436 (1.8)	1056 (2)
Insurance							
Private	42 579 (13.5)	12 990 (14.3)	6435 (14.4)	11 470 (14.7)	10 308 (14.4)	3574 (15)	7658 (14.5)
Medicaid	140 970 (44.8)	45 660 (50.3)	22 380 (50.2)	40 230 (51.5)	28 511 (39.9)	11 802 (49.4)	18 360 (34.7)
Medicare	108 140 (34.4)	24 910 (27.5)	12 504 (28.1)	20 031 (25.6)	27 237 (38.1)	5753 (24.1)	23 748 (44.8)
Other	23 074 (7.3)	7138 (7.9)	3232 (7.3)	6396 (8.2)	5434 (7.6)	2768 (11.6)	3193 (6)
Arrival method							
Ambulance	129 901 (41.3)	21 806 (24)	10 370 (23.3)	16 786 (21.5)	37 121 (51.9)	8715 (36.5)	31 948 (60.3)
Walk-in	184 862 (58.7)	68 892 (76)	34 181 (76.7)	61 341 (78.5)	34 369 (48.1)	15 182 (63.5)	21 011 (39.7)
ESI							
2	108 094 (34.3)	26 258 (29)	16 117 (36.2)	18 357 (23.5)	12 819 (17.9)	13 (0.1)	12 785 (24.1)
3	142 174 (45.2)	52 885 (58.3)	27 353 (61.4)	48 736 (62.4)	40 544 (56.7)	7735 (32.4)	36 631 (69.2)
4	64 495 (20.5)	11 555 (12.7)	1081 (2.4)	11 034 (14.1)	18 127 (25.4)	16 149 (67.6)	3543 (6.7)
High utilizer^c^							
No	259 528 (82.5)	75 529 (83.3)	36 622 (82.2)	65 474 (83.8)	59 938 (83.8)	20 737 (86.8)	43 789 (82.7)
Yes	55 235 (17.6)	15 169 (16.7)	7929 (17.8)	12 653 (16.2)	11 552 (16.2)	3160 (13.2)	9170 (17.3)

Abbreviations: ESI, emergency severity index; UQJ, unexplained queue jump.

^a^ All aggregated passed over UQJ column and receiving care ahead of others UQJ column are not the sum of the corresponding same-acuity and lower or higher-acuity columns because a patient can experience multiple types of UQJs within a single triage decision to take a patient outside of the rules of acuity based first come, first served.

^b^ Other includes American Indian or Alaska Native, Asian, Native Hawaiian, Native Hawaiian or Other Pacific Islander, and not listed.

^c^ High utilizer was a health system definition of 4 or more emergency department visits in the preceding 6 months.

### Social Factors Associated With UQJ Exposure

**Figure 1.**
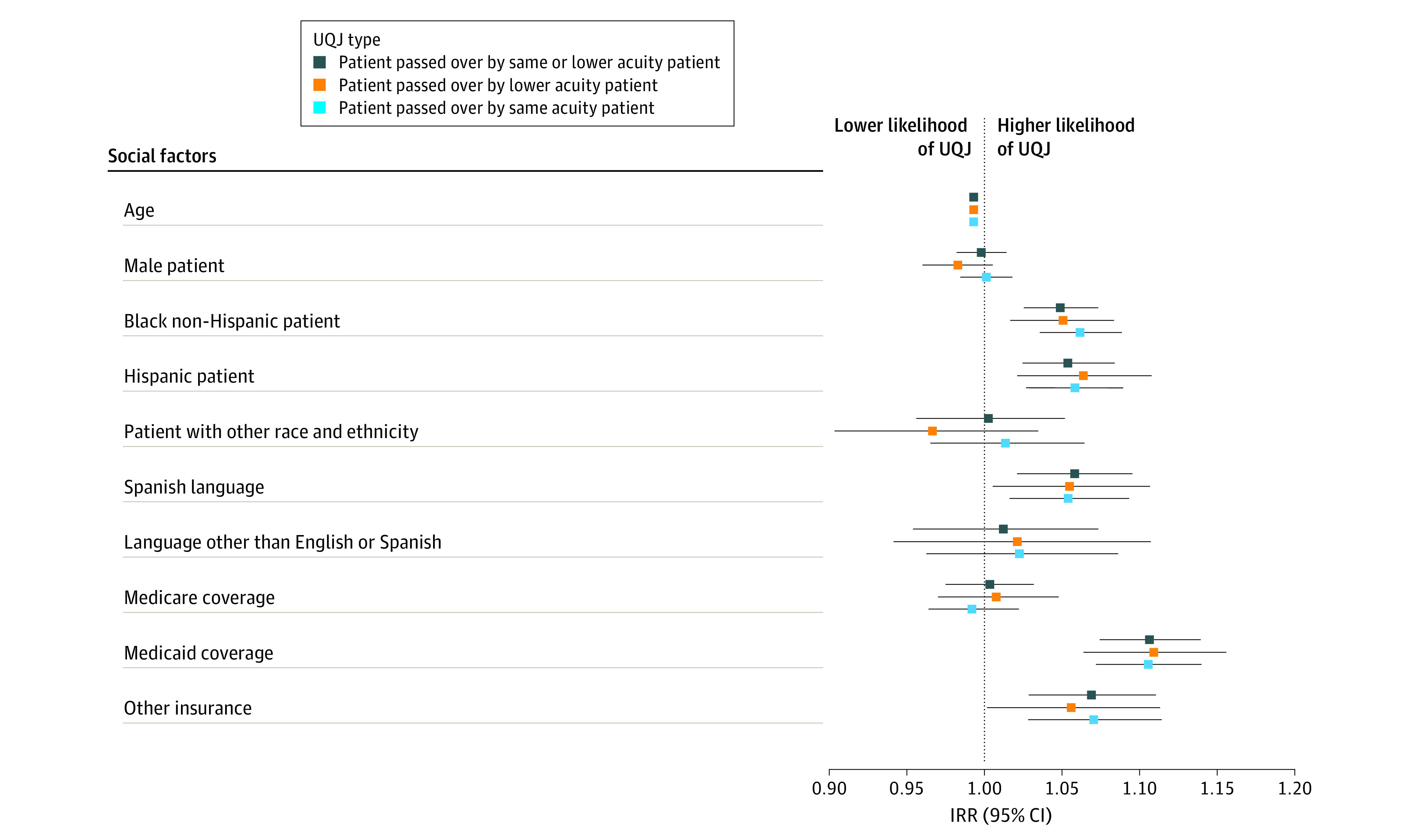
Patient Social Factors Associated With Unexplained Queue Jumping (UQJ) When a Patient is Passed Over by Another Patient Reference groups for categorical variables include female, non-Hispanic White, English language, and private insurance. IRR indicates incident rate ratio.

Patient demographics including Medicaid insurance (IRR, 1.11; 95% CI, 1.07-1.14), Black non-Hispanic race (IRR, 1.05; 95% CI, 1.03-1.07), Hispanic or Latino ethnicity (IRR, 1.05; 95% CI, 1.02-1.08), and Spanish as primary language (IRR, 1.06; 95% CI, 1.02-1.10) were independent social factors associated with being passed over. Patients with primary insurance listed as Medicaid (IRR, 1.11; 95% CI, 1.06-1.16) were more likely to be passed over by lower acuity patients compared with patients with private insurance. Similarly, patients who were non-Hispanic Black (IRR, 1.05; 95% CI, 1.02-1.08) or Hispanic (IRR, 1.06; 95% CI, 1.02-1.11) were more likely to be passed over by a lower acuity patient compared with non-Hispanic White patients. Spanish speaking patients (IRR, 1.06; 95% CI, 1.01-1.11) were more likely to be passed over by lower acuity patients compared with English speaking patients (Figure 1; eTable 4 in Supplement 1).

Results were similar for patients being passed over by same-acuity patients. Patients with their primary insurance listed as Medicaid (IRR, 1.11; 95% CI, 1.07-1.14) were more likely to be passed over by same-acuity patients compared with patients with private insurance. Patients who were non-Hispanic Black (IRR, 1.06; 95% CI, 1.04-1.09), Hispanic (IRR, 1.06; 95% CI, 1.03-1.09), or Spanish speaking (IRR, 1.05; 95% CI, 1.02-1.09) were more likely to be passed over by same-acuity patients.

**Figure 2.**
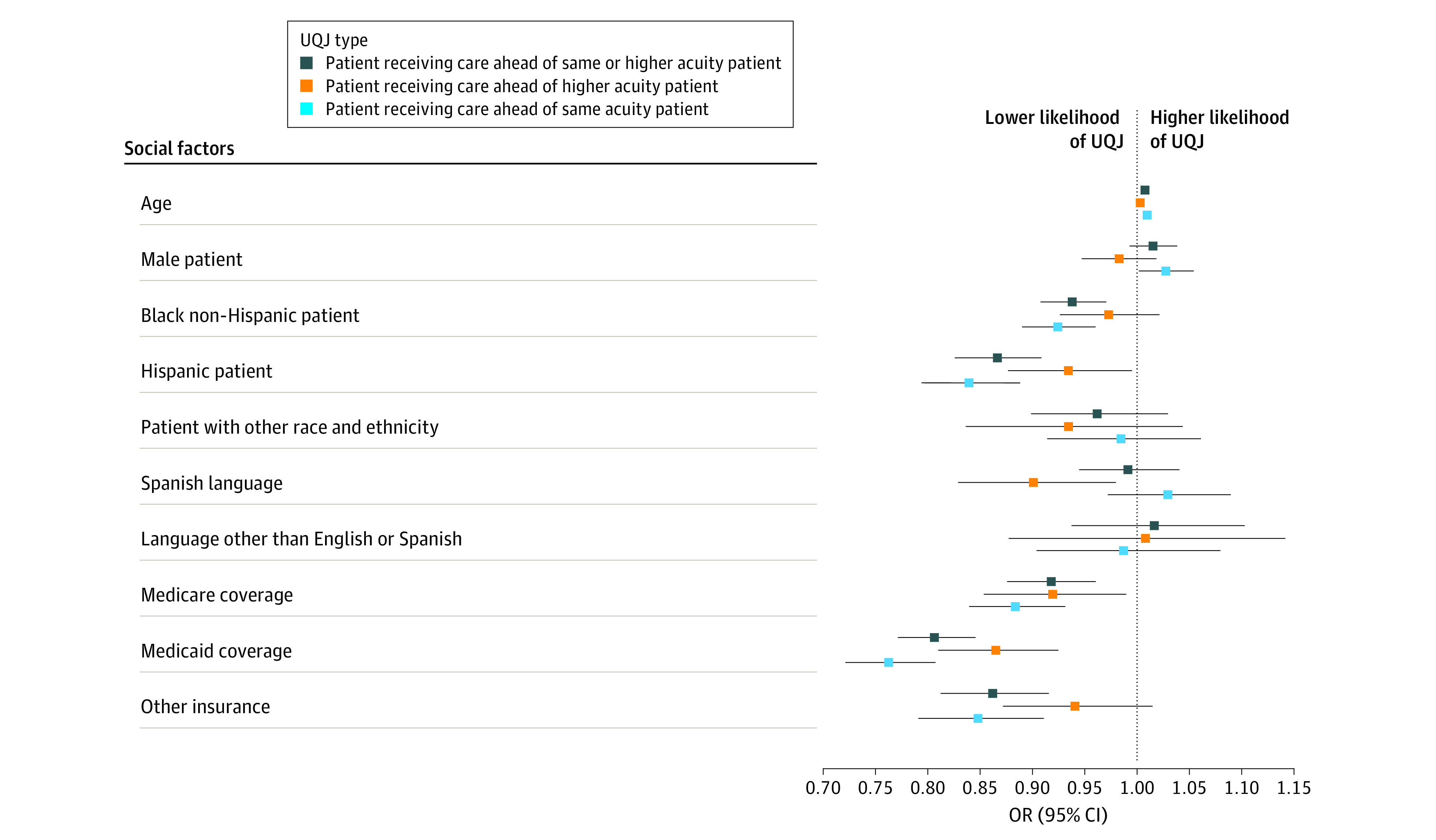
Patient Social Factors Associated With Unexplained Queue Jumping (UQJ) When a Patient is Selected to Receive Care Ahead of Another Patient Reference groups for categorical variables include female, non-Hispanic White, English language, and private insurance. OR indicates odds ratio.

The odds of a patient receiving care ahead of others were lower for ED visits by Medicare insured (OR, 0.92; 95% CI, 0.88-0.96), Medicaid insured (OR, 0.81; 95% CI, 0.77-0.85), Black non-Hispanic (OR, 0.94; 95% CI, 0.91-0.97), and Hispanic or Latino ethnicity (OR, 0.87; 95% CI, 0.83-0.91). Odds of a patient receiving care ahead of higher-acuity patients was lower for patients who had Medicare (OR, 0.92; 95% CI, 0.85-0.99) or Medicaid (OR, 0.87; 95% CI, 0.81-0.92) listed as their primary insurance as well as for Hispanic patients (OR, 0.93; 95% CI, 0.88-0.99) and Spanish speaking patients (OR, 0.90; 95% CI, 0.83-0.98). Sex was not estimated to have a statistically significant association with this outcome (Figure 2; eTable 5 in Supplement 1).

With respect to patients receiving care ahead of same-acuity patients, this outcome was higher for male patients (OR, 1.03; 95% CI, 1.00-1.05) compared with female patients. The odds of receiving care ahead of same-acuity patients were lower for patients with Medicare (OR, 0.88; 95% CI, 0.84-0.93) or Medicaid (OR, 0.76; 95% CI, 0.72-0.81) listed as their primary insurance as well as for non-Hispanic Black (OR, 0.92; 95% CI, 0.89-0.96) and Hispanic or Latino (OR, 0.84; 95% CI, 0.79-0.89) patients. Language preference was not associated with this outcome.

### Associations Between UQJ Exposure and ED Outcomes

**Figure 3.**
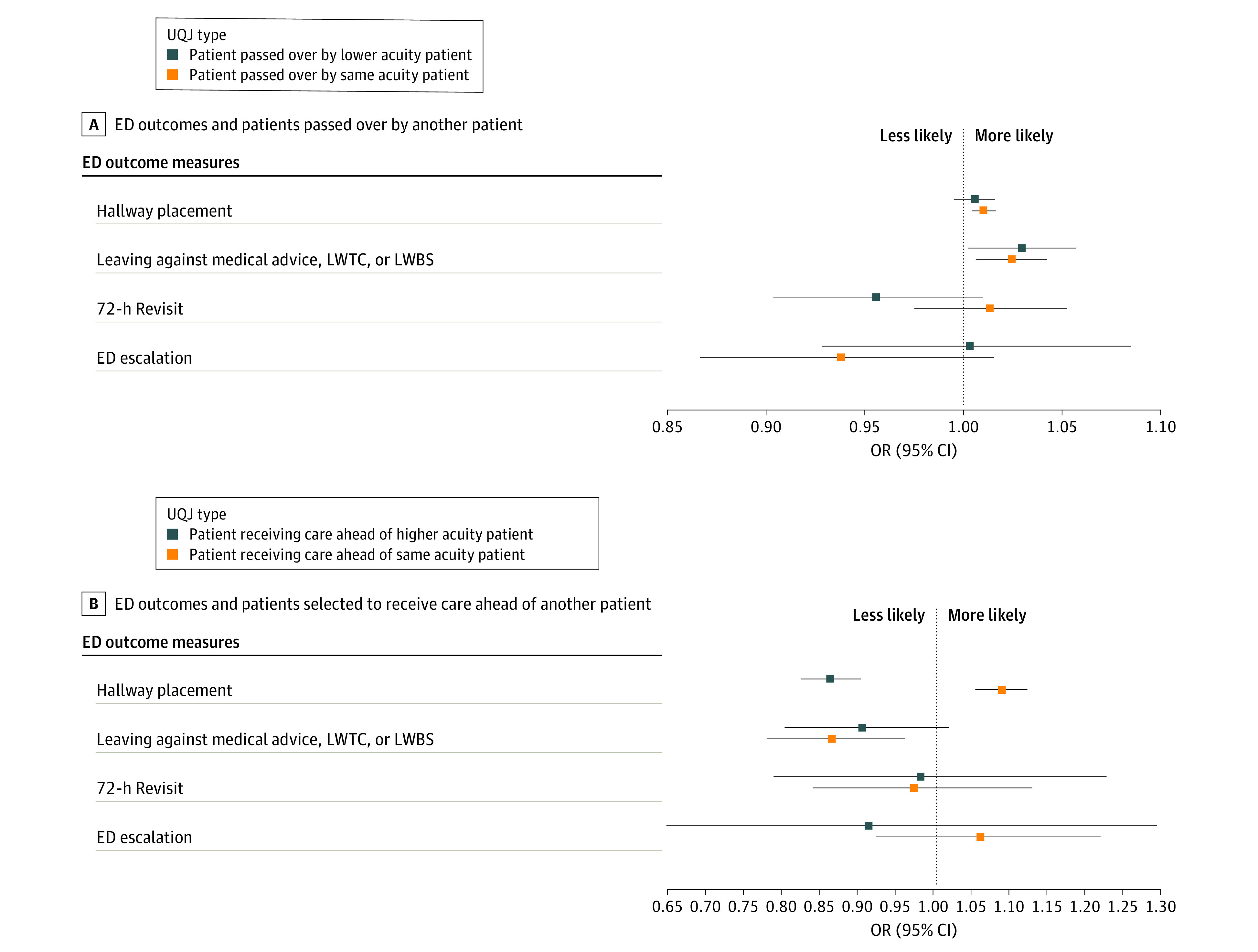
Association Between Unexplained Queue Jumps (UQJ) Exposure and Emergency Department (ED) Outcomes Reference groups are the patient population who did not experience the ED outcome of interest. LWBS indicates leaving without being seen; LWTC, leaving without treatment complete.

Patients have higher odds of leaving the ED before care completion if they experience being passed over by lower acuity patients (OR, 1.03; 95% CI, 1.00-1.06) or passed over by same-acuity patients (OR, 1.02; 95% CI, 1.01-1.04) compared with those not experiencing a UQJ (Figure 3A). Patients who were passed over by same-acuity patients had a 1.01 (95% CI, 1.00-1.02) higher odds of being placed in a hallway bed. We did not find statistically significant associations between being passed over UQJs, 72-hour ED revisits, or experiencing an escalation in admission care level while boarding.

We found patient placement in hallway beds was less likely when receiving care ahead of higher-acuity patients (OR, 0.86; 95% CI, 0.83-0.90) but higher when receiving care ahead of same-acuity patients (OR, 1.09; 95% CI, 1.06-1.12). Patients who received care ahead of same-acuity patients had 0.87 (95% CI, 0.78-0.96) lower odds of leaving the ED before care completion (Figure 3B; eTable 6 in Supplement 1).

### High Acuity Arrivals

Among the subset of 10 634 (3.4%) high acuity ED visits in our data set, we did not find statistically significant associations between patient social factors and being passed over (eFigure 3 and eTable 7 in Supplement 1). We conducted this analysis only for being passed over outcomes and not receiving care ahead outcomes since the latter are not well-defined for high acuity patients. Patients being passed over by same-acuity patients is also rare for ESI 1, but the model still converged, although with a high coefficient for ESI 2 (eTable 7 in Supplement 1).

Given the results may be sensitive to the top or bottom 1st percentile exclusion (eFigure 1 in Supplement 1), we performed a sensitivity analysis using all data and data trimmed at the 0.5th percentile. We found results to be the same with the exception that the odds of leaving before care completion (leaving against medical advice, LWTC, LWBS) for a patient passed over by a patient of lower acuity was no longer significant.

## Discussion

Our model identifies UQJs as patients who receive care ahead of others and patients passed over while waiting. We explored social disparities in ED access by examining patterns in first come, first served triage queue violations across different patient sociodemographic groups. In our analysis of front-end ED operations, we found 2 important phenomena: (1) UQJs are common and involve more than 1 in 4 ED visits and (2) UQJs may disproportionately limit access to emergency care among minoritized racial and ethnic groups, female patients, and patients who rely on Medicaid. These findings support concerns that disparities exist within traditional triage beyond chief concern, vital signs, and the subjective ESI score.

Prior work has suggested that racial disparities exist not only with access to care but also in allocation of ED treatment spaces.^5,6,24,27^ In 1 study,^24^ researchers found that patients insured by Medicaid, a proxy for low socioeconomic status, are more likely to be assigned to hallway beds instead of regular beds. Our results are congruent and build on this prior work by incorporating ED boarding volume which often results in hallway bed utilization. However, prior work has focused on operational throughput and not attempted to decipher some of the underlying mechanisms that drive prolonged wait times. Not only do we find historically marginalized groups, such as Black patients and Medicaid insured patients, experiencing more UQJs, which explains increased ED waiting, but they are also more likely to be placed in hallway beds and leave before a full evaluation.

Mechanisms of structural racism and classism could explain our observation that minoritized racial and ethnic groups and patients who were uninsured or relied on public insurance were more likely to experience disadvantageous queue violations (higher odds of being passed over and lower odds of receiving care ahead of others) than privately insured White patients. Insurance status and race and ethnicity do not inherently produce differences, nor is insurance known at time of triage; rather, they are proxies for mechanisms of racism and classism. Racism and classism operate by apportioning different degrees of systemic discrimination and privilege to patients based on their insurance plans and race and ethnicity.

Importantly, racism and classism can operate at different levels to produce the differences in queue jumping we observed. The theoretical framework from Jones et al^28^ of the levels of racism is useful for conceptualizing the mechanisms that could be driving disparities in queue jumping, including internalized, personally mediated, and institutional racism. In turn, different levels of racism operate through different stakeholders in the triaging process, including the patients, ED staff, and administrative stakeholders. Internalized racism is operant on the patient level and could manifest through differences in a patient’s propensity to self-advocate in the waiting room. Historically privileged patients, including White and privately insured patients, may be more likely to have high expectations for how expediently they receive ED services and thus advocate to be seen. Additionally, White and privately insured patients are more likely to experience rule laxity.^29,30^ Overall, this may result in these groups receiving care ahead of others, especially if they perceive they have been queue jumped given the open floor plans of the waiting rooms in this study. Contrastingly, historically disadvantaged patients, including Black and Medicaid-insured patients, may have their concerns trivialized and are less likely to self-advocate, giving them a higher likelihood of being bypassed in the queue.^29,30^

Personally mediated racism is operant through implicit biases that arise within interactions between patients and ED staff. Biases could manifest through ED staff underestimation of historically marginalized patients’ severity of illness. Prior studies have identified biases in pain scoring with Black patients receiving lower pain scores compared with White patients.^31^

Beyond interpersonal interactions in triage, mechanisms of institutional racism may also be associated with UQJs and can involve stakeholders outside the ED.^32^ For example, White and privately insured patients may be more likely to receive referral to the ED by their primary care physician who leaves a care plan note highlighting their concern or informs a specialist to expect the patient, resulting in the patient being treated earlier.^33^ By contrast, marginalized patients may have fewer medical records from long-term clinicians, have more safety alerts associated with their records, or be classified as a high utilizer for multiple ED visits.^23,34,35^ Thus, their illness severity might not be recognized at triage; indeed, studies already demonstrate that marginalized populations are more likely to be assigned lower acuity scores.^5,36^ These differences in perception of patient severity may be a cause of same-acuity queue jumping that is likely exacerbated when there is greater need for queue formation such as with ED overcrowding.^37,38,39,40^ Interestingly, in our study, we assume ESI scores are unbiased and thus our results showing higher odds of being jumped for marginalized populations may be underestimating the actual effect.

Addressing different levels of racism and classism requires a multitude of interventions ranging from instituting clinical guidelines and iterative review of queue violations to diversifying the workforce to better mirror the patient population.^41^ Our sensitivity analysis examining high acuity arrivals is fundamentally based on protocols and revealed no disparities. This suggests establishing criteria for trauma, stroke, vital signs that warrant immediate bedding overrides or remove opportunities to introduce bias and are congruent with prior work.^42^ Future work might examine how different front-end design models such as physician-in-triage, fast track, and algorithmic triage software within the EHR reduce or exacerbate unexplained queue jumping. Tools can be developed that leverage digital infrastructure to guard against bias such as clinical alerts if a queue jump has occurred and prompting the clinical team to justify it. Such transparency might help address patient complaints and/or may mitigate bias.^43^

### Limitations

This study has limitations. In this study we define queue jumping as being unexplained because we rely on observational data and do not have full information regarding why a patient was selected to receive care ahead of other patients. Reasons for queue jumping unobservable to us could include interval worsening in severity of patients’ illnesses or advocacy by patients’ family members, neither of which are recorded in the medical record. As such, some instances of queue jumping may be clinically appropriate while others are not. Second, although our work may fail to capture some residual confounding, we have attempted to minimize this risk by including a comprehensive set of control variables that capture important features of the ED at the time of patient triage and conducting our main analyses on a fairly homogeneous subset of data. Third, there could be nonrandom measurement error in collection of race and ethnicity data which is a known limitation of EHR data.^44^ Despite this, our results are consistent with prior literature and such limitations should encourage health systems to identify methods for patients to self-identify or confirm their demographic information.

## Conclusions

In this cross-sectional study of ED patients in triage, minoritized and disadvantaged patients including those insured by Medicaid, those who were Black, or those who were younger were more likely to be passed over and less likely to receive care over others despite being assigned the same triage acuity. Emergency departments should seek to standardize triage processes and protocols to mitigate conscious and unconscious biases that may be associated with patient access to emergency care.

## Notes

Supplement 1. **eFigure 1. **Study Inclusion Criteria and Sample Selection Flow Chart **eFigure 2. **Defining Unexplained Queue Jumps (UQJs) **eTable 1. **List of Control Variables Included in Regression Models **eTable 2. **Summary Statistics of Regression Control Variables **eTable 3. **Assigned ESI Level by Race and Ethnicity **eTable 4. **Regression Results for Passed Over Outcomes **eTable 5. **Regression Results for Receiving Care Ahead of Others **eTable 6. **Regression Results Showing the Association Between UQJ Exposure and ED Care Outcomes **eFigure 3. **Summary of Regression Results for Secondary Analysis on a Subset of High Acuity Patient Encounters Only **eTable 7. **Table of Regression Results for Secondary Analysis on a Subset of High Acuity Patient Encounters Only

Supplement 2.
Data Sharing Statement

## References

[1] Janke AT, Melnick ER, Venkatesh AK. Monthly rates of patients who left before accessing care in US emergency departments, 2017-2021. JAMA Netw Open. 2022;5(9):e2233708. doi:10.1001/jamanetworkopen.2022.33708 36178693 PMC9526078

[2] Venkatesh AK, Janke A, Rothenberg C, Chan E, Becher RD. National trends in emergency department closures, mergers, and utilization, 2005-2015. PLoS One. 2021;16(5):e0251729. doi:10.1371/journal.pone.0251729 34015007 PMC8136839

[3] Christ M, Grossmann F, Winter D, Bingisser R, Platz E. Modern triage in the emergency department. Dtsch Arztebl Int. 2010;107(50):892-898. doi:10.3238/arztebl.2010.0892 21246025 PMC3021905

[4] Lu FQ, Hanchate AD, Paasche-Orlow MK. Racial/ethnic disparities in emergency department wait times in the United States, 2013-2017. Am J Emerg Med. 2021;47:138-144. doi:10.1016/j.ajem.2021.03.051 33812329

[5] Schrader CD, Lewis LM. Racial disparity in emergency department triage. J Emerg Med. 2013;44(2):511-518. doi:10.1016/j.jemermed.2012.05.010 22818646

[6] Sonnenfeld N, Pitts SR, Schappert SM, Decker SL. Emergency department volume and racial and ethnic differences in waiting times in the United States. Med Care. 2012;50(4):335-341. doi:10.1097/MLR.0b013e318245a53c 22270097

[7] Hsia RY, Asch SM, Weiss RE, . Hospital determinants of emergency department left without being seen rates. Ann Emerg Med. 2011;58(1):24-32.e3. doi:10.1016/j.annemergmed.2011.01.009 21334761 PMC3126631

[8] Joseph JW, Landry AM, Kennedy M, . Association of race and ethnicity with triage emergency severity index scores and total visit work relative value units for emergency department patients. JAMA Netw Open. 2022;5(9):e2231769. doi:10.1001/jamanetworkopen.2022.31769 36103184 PMC9475380

[9] Li DR, Brennan JJ, Kreshak AA, Castillo EM, Vilke GM. Patients who leave the emergency department without being seen and their follow-up behavior: a retrospective descriptive analysis. J Emerg Med. 2019;57(1):106-113. doi:10.1016/j.jemermed.2019.03.051 31078346

[10] Sabbatini AK, Kocher KE, Basu A, Hsia RY. In-hospital outcomes and costs among patients hospitalized during a return visit to the emergency department. JAMA. 2016;315(7):663-671. doi:10.1001/jama.2016.0649 26881369 PMC8366576

[11] Smalley CM, Meldon SW, Simon EL, Muir MR, Delgado F, Fertel BS. Emergency department patients who leave before treatment is complete. West J Emerg Med. 2021;22(2):148-155. doi:10.5811/westjem.2020.11.48427 33856294 PMC7972384

[12] Janke AT, Melnick ER, Venkatesh AK. Hospital occupancy and emergency department boarding during the COVID-19 pandemic. JAMA Netw Open. 2022;5(9):e2233964. doi:10.1001/jamanetworkopen.2022.33964 36178691 PMC9526134

[13] Levin S, Toerper M, Hamrock E, . Machine-learning-based electronic triage more accurately differentiates patients with respect to clinical outcomes compared with the emergency severity index. Ann Emerg Med. 2018;71(5):565-574.e2. doi:10.1016/j.annemergmed.2017.08.005 28888332

[14] Wiler JL, Gentle C, Halfpenny JM, . Optimizing emergency department front-end operations. Ann Emerg Med. 2010;55(2):142-160.e1. doi:10.1016/j.annemergmed.2009.05.021 19556030

[15] Wuerz R, Fernandes CMB, Alarcon J; Emergency Department Operations Research Working Group. Inconsistency of emergency department triage. Ann Emerg Med. 1998;32(4):431-435. doi:10.1016/S0196-0644(98)70171-4 9774926

[16] Franklin BJ, Li KY, Somand DM, . Emergency department provider in triage: assessing site-specific rationale, operational feasibility, and financial impact. J Am Coll Emerg Physicians Open. 2021;2(3):e12450. doi:10.1002/emp2.12450 34085053 PMC8144283

[17] Yiadom MYAB, Napoli A, Granovsky M, . Managing and measuring emergency department care: results of the fourth emergency department benchmarking definitions summit. Acad Emerg Med. 2020;27(7):600-611. doi:10.1111/acem.13978 32248605

[18] Batt RJ, Terwiesch C. Waiting patiently: an empirical study of queue abandonment in an emergency department. Manage Sci. 2015;61(1):39-59. doi:10.1287/mnsc.2014.2058

[19] Ding Y, Park E, Nagarajan M, Grafstein E. Patient prioritization in emergency department triage systems: an empirical study of the Canadian Triage and Acuity Scale (CTAS). Manuf Serv Oper Manag. 2019;21(4):723-741. doi:10.1287/msom.2018.0719

[20] Akşin Z, Ata B, Emadi SM, Su C-L. Impact of delay announcements in call centers: an empirical approach. Oper Res. 2016;65(1):242-265. doi:10.1287/opre.2016.1542

[21] Lu Y, Musalem A, Olivares M, Schilkrut A. Measuring the effect of queues on customer purchases. Manage Sci. 2013;59(8):1743-1763. doi:10.1287/mnsc.1120.1686

[22] Khidir H, Salhi R, Sabbatini AK, . A quality framework to address racial and ethnic disparities in emergency department care. Ann Emerg Med. 2022;81(1):47-56. doi:10.1016/j.annemergmed.2022.08.419 36257864 PMC9780164

[23] National Institutes of Health Office of Research on Women’s Health. NIH inclusion outreach toolkit: how to engage, recruit, and retain women in clinical research. 2022. Accessed Jun 23, 2023. https://orwh.od.nih.gov/toolkit/other-relevant-federal-policies/OMB-standards

[24] Kim DA, Sanchez LD, Chiu D, Brown IP. Social determinants of hallway bed use. West J Emerg Med. 2020;21(4):949-958. doi:10.5811/westjem.2020.4.45976 32726269 PMC7390564

[25] Wachelder JJH, van Drunen I, Stassen PM, . Association of socioeconomic status with outcomes in older adult community-dwelling patients after visiting the emergency department: a retrospective cohort study. BMJ Open. 2017;7(12):e019318. doi:10.1136/bmjopen-2017-019318 29282273 PMC5770947

[26] R Project for Statistical Computing. R: A language and environment for statistical computing. 2020. Accessed June 27, 2023. https://www.rstudio.com/

[27] Banco D, Chang J, Talmor N, . Sex and race differences in the evaluation and treatment of young adults presenting to the emergency department with chest pain. J Am Heart Assoc. 2022;11(10):e024199. doi:10.1161/JAHA.121.024199 35506534 PMC9238573

[28] Jones CP. Levels of racism: a theoretic framework and a gardener’s tale. Am J Public Health. 2000;90(8):1212-1215. doi:10.2105/AJPH.90.8.1212 10936998 PMC1446334

[29] Jones CP, Truman BI, Elam-Evans LD, . Using “socially assigned race” to probe white advantages in health status. Ethn Dis. 2008;18(4):496-504. 19157256

[30] Kwate NO, Goodman MS. An empirical analysis of White privilege, social position and health. Soc Sci Med. 2014;116(116):150-160. doi:10.1016/j.socscimed.2014.05.041 25014267 PMC4157125

[31] Hoffman KM, Trawalter S, Axt JR, Oliver MN. Racial bias in pain assessment and treatment recommendations, and false beliefs about biological differences between blacks and whites. Proc Natl Acad Sci U S A. 2016;113(16):4296-4301. doi:10.1073/pnas.1516047113 27044069 PMC4843483

[32] Blanchard JC, Haywood YC, Scott C. Racial and ethnic disparities in health: an emergency medicine perspective. Acad Emerg Med. 2003;10(11):1289-1293. doi:10.1111/j.1553-2712.2003.tb00615.x 14597507

[33] Landon BE, Onnela J-P, Meneades L, O’Malley AJ, Keating NL. Assessment of racial disparities in primary care physician specialty referrals. JAMA Netw Open. 2021;4(1):e2029238. doi:10.1001/jamanetworkopen.2020.29238 33492373 PMC7835717

[34] Agarwal AK, Seeburger E, O’Neill G, . Prevalence of behavioral flags in the electronic health record among black and white patients visiting the emergency department. JAMA Netw Open. 2023;6(1):e2251734. doi:10.1001/jamanetworkopen.2022.51734 36656576 PMC9857105

[35] Parast L, Mathews M, Martino S, Lehrman WG, Stark D, Elliott MN. Racial/ethnic differences in emergency department utilization and experience. J Gen Intern Med. 2022;37(1):49-56. doi:10.1007/s11606-021-06738-0 33821410 PMC8021298

[36] Vigil JM, Alcock J, Coulombe P, . Ethnic disparities in emergency severity index scores among U.S. veteran’s affairs emergency department patients. PLoS One. 2015;10(5):e0126792. doi:10.1371/journal.pone.0126792 26024515 PMC4449190

[37] Sabin JA, Rivara FP, Greenwald AG. Physician implicit attitudes and stereotypes about race and quality of medical care. Med Care. 2008;46(7):678-685. doi:10.1097/MLR.0b013e3181653d58 18580386

[38] Stepanikova I. Racial-ethnic biases, time pressure, and medical decisions. J Health Soc Behav. 2012;53(3):329-343. doi:10.1177/0022146512445807 22811465

[39] Wigboldus DHJ, Sherman JW, Franzese HL, Knippenberg Av. Capacity and comprehension: spontaneous stereotyping under cognitive load. Soc Cogn. 2004;22(3):292-309. doi:10.1521/soco.22.3.292.35967

[40] Burgess DJ. Are providers more likely to contribute to healthcare disparities under high levels of cognitive load? How features of the healthcare setting may lead to biases in medical decision making. Med Decis Making. 2010;30(2):246-257. doi:10.1177/0272989X09341751 19726783 PMC3988900

[41] Richardson LD, Babcock Irvin C, Tamayo-Sarver JH. Racial and ethnic disparities in the clinical practice of emergency medicine. Acad Emerg Med. 2003;10(11):1184-1188. doi:10.1111/j.1553-2712.2003.tb00601.x 14597493

[42] Madsen TE, Choo EK, Seigel TA, Palms D, Silver B. Lack of gender disparities in emergency department triage of acute stroke patients. West J Emerg Med. 2015;16(1):203-209. doi:10.5811/westjem.2014.11.23063 25671042 PMC4307718

[43] Sabin JA. Tackling implicit bias in health care. N Engl J Med. 2022;387(2):105-107. doi:10.1056/NEJMp2201180 35801989 PMC10332478

[44] Polubriaginof FCG, Ryan P, Salmasian H, . Challenges with quality of race and ethnicity data in observational databases. J Am Med Inform Assoc. 2019;26(8-9):730-736. doi:10.1093/jamia/ocz113 31365089 PMC6696496

