# Supplementary material for: Sociodemographic Disparities in Queue Jumping for Emergency Department Care
Source: JAMA Netw Open. 2023 Jul 28;6(7):e2326338. doi: 10.1001/jamanetworkopen.2023.26338 (PMC10383013; doi:10.1001/jamanetworkopen.2023.26338)
Supplement: Supplement 1. — eFigure 1. Study Inclusion Criteria and Sample Selection Flow Chart eFigure 2. Defining Unexplained Queue Jumps (UQJs) eTable 1. List of Control Variables Included in Regression Models eTable 2. Summary Statistics of Regression Control Variables eTable 3. Assigned ESI Level by Race and Ethnicity eTable 4. Regression Results for Passed Over Outcomes eTable 5. Regression Results for Receiving Care Ahead of Others eTable 6. Regression Results Showing the Association Between UQJ Exposure and ED Care Outcomes eFigure 3. Summary of Regression Results for Secondary Analysis on a Subset of High Acuity Patient Encounters Only eTable 7. Table of Regression Results for Secondary Analysis on a Subset of High Acuity Patient Encounters Only [file jamanetwopen-e2326338-s001.pdf]

# Supplemental Online Content

Sangal RB, Su H, Khidir H. Sociodemographic disparities in queue jumping for emergency department care. *JAMA Netw Open*. 2023;6(7):e2326338. doi:10.1001/jamanetworkopen.2023.26338

**eFigure 1.** Study Inclusion Criteria and Sample Selection Flow Chart
**eFigure 2.** Defining Unexplained Queue Jumps (UQJs)
**eTable 1.** List of Control Variables Included in Regression Models
**eTable 2.** Summary Statistics of Regression Control Variables
**eTable 3.** Assigned ESI Level by Race and Ethnicity
**eTable 4.** Regression Results for Passed Over Outcomes
**eTable 5.** Regression Results for Receiving Care Ahead of Others
**eTable 6.** Regression Results Showing the Association Between UQJ Exposure and ED Care Outcomes
**eFigure 3.** Summary of Regression Results for Secondary Analysis on a Subset of High Acuity Patient Encounters Only
**eTable 7.** Table of Regression Results for Secondary Analysis on a Subset of High Acuity Patient Encounters Only

This supplemental material has been provided by the authors to give readers additional information about their work.

© 2023 Sangal RB et al. *JAMA Network Open.*

**eFigure 1**. Study Inclusion Criteria and Sample Selection Flow Chart

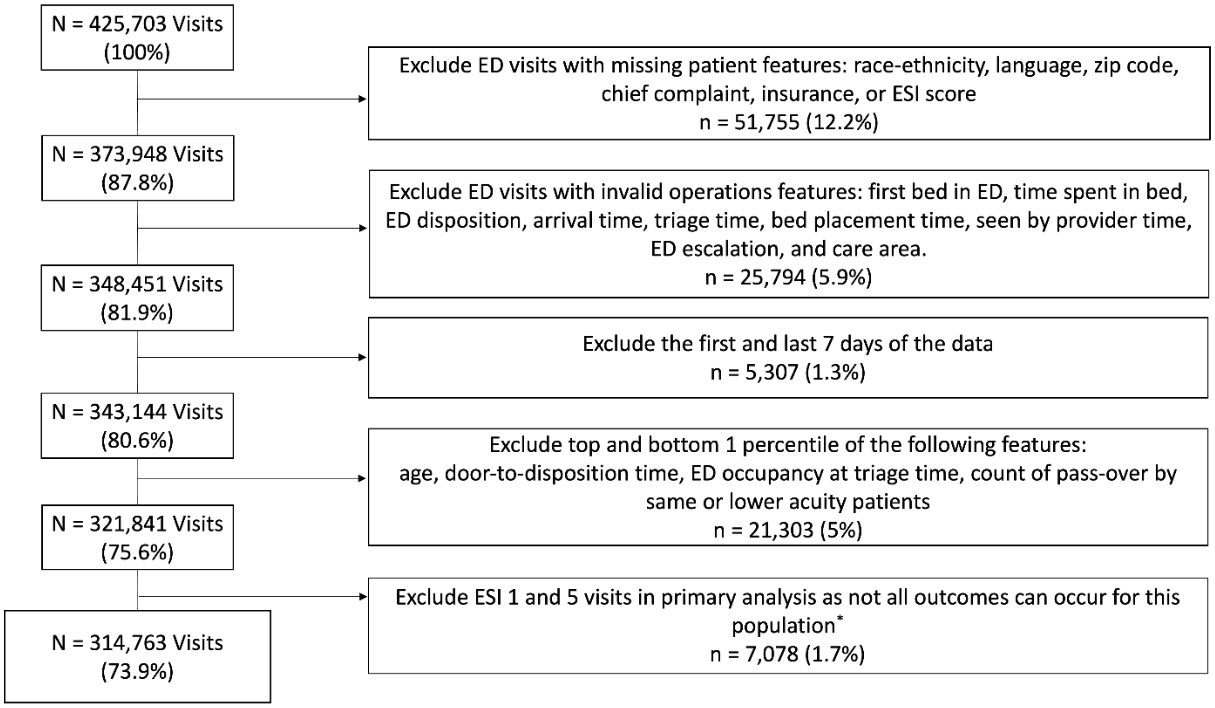


*Being passed over by lower acuity patient cannot occur to ESI-5 visits since this is the lowest acuity score. Similarly, receiving care ahead of higher acuity patients cannot occur to ESI-1 visits since this is the highest acuity score. All patients are included in census control variables.
** We twice repeated our analyses with (1) no data exclusions and (2) excluding the top and bottom 0.5 percentile. We find qualitatively identical results, except for our estimate on the association between patients who were passed over by a patient of lower acuity on the probability that the patient experienced a premature departure (AMA, LWTC, LWBS). Using the 1 percentile trim as described in this figure, the result is significant at the 0.05 level, but is not significant when we either use all data, or trim only the top and bottom 0.5 percentile.

© 2023 Sangal RB et al. *JAMA Network Open.*

**eFigure 2**. Defining Unexplained Queue Jumps (UQJs). Illustrations of patient routing from the waiting room to an ED treatment bed. Panel I (top) illustrates acuity based first-come, first-served. Panel II (bottom) illustrates unexplained queue jumping (UQJ) instances.

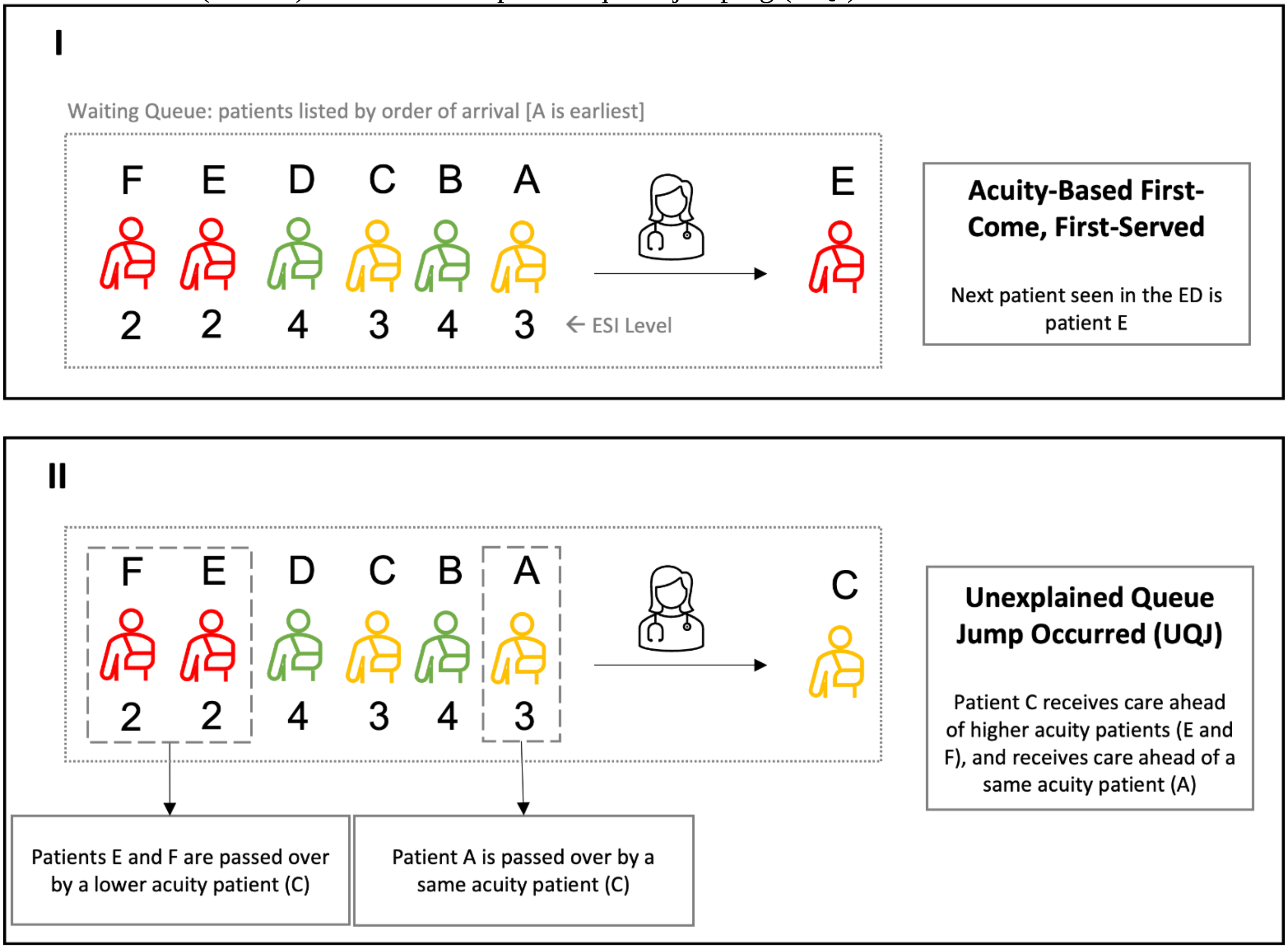


© 2023 Sangal RB et al. *JAMA Network Open.*

**eTable 1.** List of Control Variables Included in Regression Models

| | Variables used for all outcome | | |
|---|---|---|---|
| **Category** | **Variables** | **Primary Analysis Usage** | **Sensitivity Analysis Usage** |
| Social Factor | Age, Sex, Race-Ethnicity, Insurance, Language | Independent variable | Control variable |
| ED Encounter | Chief Complaint Type**, ESI Level, Arrival Method, Transfer In, Reevaluation, High-Utilizer, Home Zip Code, ED Location | Control variable | Control variable |
| ED Staffing* | Nurse-to-patient ratio, Physician-to-patient ratio, Resident-to-patient ratio | Control variable | Control variable |
| Time/Day | Year, Month, Time of day, Day of week, Holiday (Binary) | Control variable | Control variable |
| ED Occupancy* | Number of patients in ED hallway bed, Number of patients in ED regular bed, Number of patients in the waiting room, Number of patients boarding | Control variable | Control variable |
| ED Patient Mixture* | Average ESI for hallway patients, regular-bed patients, and all patients, Variance of ESI for hallway patients, regular-bed patients, and all patients, Number of unique chief complaints in the waiting room, Number of patients who have the same chief complaint as the index patient, Average ESI in the waiting room, the variance of ESI in the waiting room | Control variable | Control variable |

******Calculated at the triage time of the index encounter*

*** To keep the ED visit population homogeneous, we followed previous studies[1] and grouped 701 chief complaints into 29 categories: Abdominal symptoms, Back and neck pain, Cardiac, Device-related, Chest pain, Cold / Fever / Infection, Dermatologic, Deviating lab values, Dizziness/syncope, Extremity injury, Extremity symptoms without injury, Eye/ear/nose/throat problems, Dental, Head and face symptoms, Fall, Focal neurological symptom, GI bleeding, Rectal disorders, Nausea, vomiting and diarrhea, Genito-urinary problem, Headache, Medication/procedure, Gynecological problem, Psychiatric/social problems, Respiratory problems, Substance abuse, Traffic injury, Traumatic and Unwell.*

| Additional Variables used for different secondary outcomes | | |
|---|---|---|
| | **Variables** | **Usage** |
| 1 | Received care ahead of higher acuity patients, Received care ahead of same acuity patients, Passed-over(s) by lower acuity patients, Passed-over(s) by same acuity patients | As independent variable in all secondary outcomes |
| 2 | Hallway placement (Binary), Door-to-room time | As control variable in<br>2.2 Abnormal departure after bed placement<br>2.3 Revisit outcomes |
| 3 | Room-to-departure, number of radiology orders | As control variable in 2.3 Revisit outcomes |
| 4 | Door-to-doctor, doctor-to-disposition | As control variable in 2.4 Downgrade/Escalation during boarding |

[1] https://www.sciencedirect.com/science/article/pii/S0099176708002584?via%3Dihub
https://tsaco.bmj.com/content/tsaco/6/1/e000604.full.pdf
https://onlinelibrary.wiley.com/doi/pdf/10.1111/j.1553-2712.2001.tb01098.x

© 2023 Sangal RB et al. *JAMA Network Open.*

**eTable 2.** Summary Statistics of Regression Control Variables

| Category | Control Variable | Summary Statistic | |
|---|---|---|---|
| Patient Encounter Level | Insurance Type | Private: 13.4% // Medicaid: 45% // Medicare: 34.2% // Other: 7.4% | |
| | Age | Mean: 50.312// SD: 20.48 // Min: 9 // Max: 94 | |
| | Sex | Female: 53.9%// Male: 46.1% | |
| | Race-Ethnicity | Black non-Hispanic: 34% // Hispanic or Latino: 21.1% // White non-Hispanic: 42% // Other*: 2.9% | |
| | Address Zip Code | 06510: 2% // 06405: 2.8% // 06437: 1% // 06460: 1.4% // 06473: 2.6% // 06477: 1% // 06492: 1.9% // 06511: 17.1% // 06512: 5.5% // 06513: 12% // 06514: 6% // 06515: 4.6% // 06516: 9.9% // 06517: 3% // 06518: 1.8% // 06519: 7.4% // Others: 19.8% | |
| | ED Arrival Method | Ambulance: 41.1% // Walk In: 58.9% | |
| | Acuity – ESI Level | 1: 0.6% // 2: 33.6% // 3: 44.2% // 4: 20% // 5: 1.6% | |
| | Preferred Language | English: 90% // Other: 1.8% // Spanish: 8.1% | |
| | Transfer Patient | Yes: 4% // No: 96% | |
| | High ED Utilizer | Yes: 17.5% // No: 82.5% | |
| | Chief Complaint | Abdominal symptoms : 10.5%// Back and neck pain: 3.7% // Cardiac: 1.1% // Chest pain : 6.9% // Cold / Fever / Infection: 2.9% // Dental: 1% // Dermatologic: 5.1% // Deviating lab values : 3.3% // Device-related: 0.8% // Dizziness/syncope: 3.7% // Extremity injury: 1.9% // Extremity symptoms without injury: 7.9% // Eye/ear/nose/throat problems: 3.7% // Fall: 4.7% // Female Genito-urinary problem: 2% // Focal neurological symptom : 5.3% // GI bleeding : 0.9% // Gynecological problem: 1% // Headache: 2.4% // Male Genito-urinary problem: 1.3% // Medication/procedure: 3.5% // Nausea, vomiting and diarrhea: 2.6% // Others: 0.6% // Psychiatric/social problems: 4.8% // Respiratory problems: 8.1% // Substance abuse: 5.2% // Traffic injury: 3.3% // Traumatic: 0.5% // Unwell : 1.2% | |
| Date-Time Level | Time of Day (0-23) | 0: 3.2%// 1: 2.7% // 2: 2.2% // 3: 2% // 4: 1.6% // 5: 1.5% // 6: 1.7% // 7: 2.3% // 8: 3.3% // 9: 4.9% // 10: 5.1% // 11: 7.8% // 12: 6.1% // 13: 5.8% // 14: 5.7% // 15: 5.7% // 16: 5.7% // 17: 5.7% // 18: 5.5% // 19: 5.1% // 20: 4.9% // 21: 4.4% // 22: 3.7% // 23: 3.5% | |
| | Day of Week | Monday: 14.9% // Tuesday: 14.6% // Wednesday: 14.4% // Thursday: 14.2% // Friday: 14.5% // Saturday: 13.8% // Sunday: 13.5% | |
| | Month | Jan: 9.5% // Feb: 8.1% // Mar: 6.3% // Apr: 6.2% // May: 6.6% // Jun: 6.4% // Jul: 9.1% // Aug: 10% // Sep: 9.6% // Oct: 9.7% // Nov: 9.1% // Dec: 9.3% | |
| | Year | 2017: 18.4% // 2018: 38.6% // 2019: 37.5% // 2020: 5.4% | |
| | Holiday | Yes: 0.8% // No: 99.2% | |
| ED Level | ED Location | ED Location 1: 41.9% // ED Location 2: 58.1% | |
| | ED Occupancy[1] | Regular beds occupied: | Mean: 49.7// SD: 18.78 // Min: 8 // Max: 97 |
| | | Hallway beds occupied: | Mean: 11.2// SD: 8.8 // Min: 0 // Max: 46 |
| | | The number of boarding patients in the ED | Mean: 16.1// SD: 11.8 // Min: 0 // Max: 80 |
| | | The number of patients in the waiting room. | Mean: 5.2// SD: 4.98 // Min: 0 // Max: 39 |

© 2023 Sangal RB et al. *JAMA Network Open.*

| | | |
|---|---|---|
| ED Patient Mix | Number of unique chief complaints in the waiting room at the index patient's triage time | Mean: 4.6// SD: 4.1 // Min: 0 // Max: 29 |
| | Number of patients in the waiting room who have the same chief complaints as the index patient at the index patient's triage time | Mean: 1// SD: 0.55 // Min: 0 // Max: 12 |
| | Average ESI score for patients who have been moved to a bed in the ED at the index patient's triage time (Regular and Hall) | |
| | Variance of ESI score for patients who have been moved to a bed in the ED at the index patient's triage time. (Regular and Hall) | Mean: 2.562// SD: 0.2 // Min: 2 // Max: 3.429 |
| | | Mean: 2.57// SD: 0.572 // Min: 2 // Max: 5 |
| | | Mean: 0.403// SD: 0.119 // Min: 0.062 // Max: 1.306 |
| | | Mean: 0.383// SD: 0.281 // Min: 0 // Max: 3.04 |
| ED Staffing Level | Patient-Nurse Ratio | Mean: 2.97// SD: 0.645 // Min: 0.941 // Max: 7.1 |
| | Patient-Resident Ratio | Mean: 6.48// SD: 1.7 // Min: 1.5 // Max: 17 |
| | Patient-Physician Ratio | Mean: 11.9// SD: 7.3 // Min: 2 // Max: 43.5 |

*Race-Ethnicity "Other": 2.9% -
American Indian or Alaska Native: 0.27%
Asian: 1.3%
Native Hawaiian or Other Pacific Islander: 0.062%
Not Listed : 1.2%

**eTable 3 Assigned ESI Level by Race and Ethnicity**

| | Black Non-Hispanic | Hispanic or Latino | White Non-Hispanic | Other* | |
|---|---|---|---|---|---|
| ESI | 109,546 | 67,912 | 135,125 | 9,258 | X2=19,795.9 *** |
| 1 | 487 (0.4%) | 236 (0.3%) | 1,273 (0.9%) | 39 (0.4%) | |
| 2 | 29,698 (27.1%) | 15,732 (23.2%) | 59,938 (44.4%) | 2,726 (29.4%) | |
| 3 | 46,835 (42.8%) | 32,962 (48.5%) | 58,029 (42.9%) | 4,348 (47%) | |
| 4 | 29,868 (27.3%) | 17,771 (26.2%) | 14,846 (11%) | 2,010 (21.7%) | |
| 5 | 2,658 (2.4%) | 1,211 (1.8%) | 1,039 (0.8%) | 135 (1.5%) | |

*** p < 0.001
** Other Race-Ethnicity:*
*American Indian or Alaska Native, Asian, Native Hawaiian, Native Hawaiian or Other Pacific Islander and Not Listed*

© 2023 Sangal RB et al. *JAMA Network Open.*

**eTable 4.** Regression Results for Passed Over Outcomes
The first column is the regression result for the count of passed over by same or lower acuity patients, the second column is the regression result for the count of passed over by lower acuity patients, the third column is the regression result for the count of passed over by same acuity patients. Coefficients presented are the incidence rate ratio (or relative risk), where values above 1.0 represent an increase in the incidence rate, and values below 1.0 represent a decrease in the incidence rate.

## Social Risk Factors for Getting Passed Over

| | *Dependent variable:* | | |
|---|---|---|---|
| | *Passed over by same or lower acuity patients* | *Passed over by lower acuity patients* | *Passed over by same acuity patients* |
| | *IRR (95%CI LB,UB)* | *IRR (95%CI LB,UB)* | *IRR (95%CI LB,UB)* |
| | *(1)* | *(2)* | *(3)* |
| Age | 0.99327***(0.99244,0.99411) | 0.99321***(0.99205,0.99437) | 0.99333***(0.99246,0.99419) |
| Arrival Method: Walk-In (ambulance as baseline) | 3.29521***(3.18456,3.40969) | 3.40447***(3.24396,3.57292) | 3.13334***(3.02354,3.24711) |
| High Utilizer | 1.05351***(1.02858,1.07903) | 1.03457*(1.00312,1.067) | 1.0555***(1.02903,1.08265) |
| Sex: Male (Female baseline) | 0.99844(0.98261,1.01453) | 0.98286(0.96049,1.00575) | 1.00167(0.98536,1.01824) |
| ESI3(ESI 2 as baseline) | 1.72804***(1.66703,1.79128) | 1.05533***(1.01975,1.09214) | 2.43689***(2.34672,2.53052) |
| ESI4(ESI 2 as baseline) | 0.27888***(0.26433,0.29424) | 0.03841***(0.03419,0.04314) | 0.49908***(0.47241,0.52726) |
| Transfer In: Yes | 0.78425***(0.73767,0.83379) | 0.83901***(0.77238,0.91137) | 0.74516***(0.69503,0.79892) |
| Race-Ethnicity: Black non-Hispanic (White non-Hispanic as baseline) | 1.04951***(1.02566,1.0739) | 1.05024**(1.01727,1.08428) | 1.06214***(1.03599,1.08894) |
| Race-Ethnicity: Hispanic or Latino (White non-Hispanic as baseline) | 1.05427***(1.02481,1.08458) | 1.06416**(1.02189,1.10818) | 1.05822***(1.02725,1.09013) |
| Race-Ethnicity: Others* (White non-Hispanic as baseline) | 1.00307(0.95622,1.05221) | 0.96691(0.9034,1.03489) | 1.01369(0.96522,1.0646) |
| Insurance: Medicaid (private insurance as baseline) | 1.10679***(1.07451,1.14003) | 1.10937***(1.06428,1.15638) | 1.10591***(1.07274,1.14012) |
| Insurance: Medicare (private insurance as baseline) | 1.00352(0.97539,1.03246) | 1.00843(0.97024,1.04813) | 0.99283(0.9641,1.02241) |
| Insurance: Other (private insurance as baseline) | 1.06939***(1.0291,1.11126) | 1.05629*(1.00207,1.11345) | 1.07076***(1.02874,1.1145) |
| Language: Other (English as baseline) | 1.01257(0.95447,1.07421) | 1.02124(0.94159,1.10763) | 1.02268(0.96282,1.08627) |
| Language: Spanish (English as baseline) | 1.05833***(1.02157,1.09641) | 1.05506*(1.00559,1.10695) | 1.05441**(1.01658,1.09364) |
| ED Level Controls: | YES | YES | YES |
| Staffing Level Control | YES | YES | YES |
| Patient mixture Control | YES | YES | YES |

© 2023 Sangal RB et al. *JAMA Network Open.*

| | | | |
|---|---|---|---|
| Day of Week Control (factor) | YES | YES | YES |
| Time of Day Control (hour, factor) | YES | YES | YES |
| Year Control (year, factor) | YES | YES | YES |
| Patient Residential Zipcode Control (factor) | YES | YES | YES |
| Patient Chief Complaint Control (factor) | YES | YES | YES |
| Constant | 0.23795***(0.17264,0.32796) | 0.45233***(0.29486,0.6939) | 0.11136***(0.07718,0.16068) |
| Observations | 314,763 | 314,763 | 314,763 |
| Log Likelihood | -329,351.70000 | -165,094.40000 | -275,001.30000 |

*Note:* + p<0.1; * p<0.05; ** p<0.01; *** p<0.001

** Other Race-Ethnicity:*
*American Indian or Alaska Native, Asian, Native Hawaiian, Native Hawaiian or Other Pacific Islander and Not Listed*

**eTable 5** Regression Results for Receiving Care Ahead of Others
The first column shows the regression results for receiving care ahead of same or higher acuity patients, the second column shows the regression result for receiving care ahead of higher acuity patients, the third column is the regression result for receiving care ahead of same acuity patients. Coefficients presented are odds ratios where values below 1.0 represent a decrease in the odds, and values above 1.0 represent an increase in the odds.

### Social Risk Factors for Receiving Care Ahead

| | *Dependent variable:* | | |
|---|---|---|---|
| | *Received care ahead of same or higher acuity patients* | *Received care ahead of higher acuity patients* | *Received care ahead of same acuity patients* |
| | *OR (95%CI LB,UB)* | *OR (95%CI LB,UB)* | *OR (95%CI LB,UB)* |
| | *(1)* | *(2)* | *(3)* |
| Age | 1.00712***(1.00601,1.00825) | 1.00349***(1.0019,1.00508) | 1.00903***(1.00769,1.01037) |
| Arrival Method: Walk-In (ambulance as baseline) | 0.27285***(0.26036,0.28593) | 0.37344***(0.34575,0.40335) | 0.25689***(0.24416,0.27029) |
| High Utilizer | 0.95394**(0.92325,0.98566) | 0.90308***(0.84977,0.95974) | 0.98873(0.95693,1.02158) |
| Sex: Male (Female baseline) | 1.01504(0.99237,1.03822) | 0.98208(0.94677,1.0187) | 1.02693*(1.00045,1.05412) |
| ESI3(ESI 2 as baseline) | 12.51965***(11.83131,13.24804) | 1609.05093***(681.05641,3801.51311) | 9.37039***(8.84482,9.92719) |
| ESI4(ESI 2 as baseline) | 22.16517***(20.58492,23.86672) | 27615.26897***(11169.12522,68277.7805) | 1.45206***(1.34126,1.57202) |
| Transfer In: Yes | 1.03526(0.98085,1.09268) | 1.15685*(1.02916,1.30037) | 0.98314(0.93189,1.0372) |

© 2023 Sangal RB et al. *JAMA Network Open.*

| | | | |
|---|---|---|---|
| Race-Ethnicity: Black non-Hispanic (White non-Hispanic as baseline) | 0.93821***(0.9075,0.96997) | 0.97252(0.92637,1.02098) | 0.92436***(0.88996,0.96008) |
| Race-Ethnicity: Hispanic or Latino (White non-Hispanic as baseline) | 0.86658***(0.82605,0.9091) | 0.93433*(0.87698,0.99544) | 0.83937***(0.79355,0.88784) |
| Race-Ethnicity: Others (White non-Hispanic as baseline) | 0.96189(0.89834,1.02994) | 0.93399(0.83583,1.04368) | 0.98442(0.91396,1.06032) |
| Insurance: Medicaid (private insurance as baseline) | 0.80721***(0.77068,0.84548) | 0.8653***(0.80966,0.92477) | 0.76268***(0.72091,0.80687) |
| Insurance: Medicare (private insurance as baseline) | 0.91668***(0.87576,0.95952) | 0.91924*(0.85377,0.98973) | 0.88373***(0.8385,0.9314) |
| Insurance: Other (private insurance as baseline) | 0.86221***(0.81158,0.916) | 0.94088(0.87195,1.01526) | 0.84884***(0.79142,0.91042) |
| Language: Other (English as baseline) | 1.01636(0.9362,1.10339) | 1.00208(0.87629,1.14592) | 0.98749(0.90344,1.07935) |
| Language: Spanish (English as baseline) | 0.99119(0.94422,1.04049) | 0.90142*(0.82948,0.97959) | 1.02899(0.97176,1.0896) |
| ED Level Controls: | YES | YES | YES |
| Staffing Level Control | YES | YES | YES |
| Patient mixture Control | YES | YES | YES |
| Day of Week Control (factor) | YES | YES | YES |
| Time of Day Control (hour, factor) | YES | YES | YES |
| Year Control (year, factor) | YES | YES | YES |
| Patient Residential Zipcode Control (factor) | YES | YES | YES |
| Patient Chief Complaint Control (factor) | YES | YES | YES |
| Constant | 0.06005***(0.03851,0.09365) | 0.00063***(0.00021,0.00184) | 0.02358***(0.01359,0.0409) |
| Observations | 314,763 | 314,763 | 314,763 |
| Log Likelihood | -114,360.90000 | -47,827.58000 | -95,157.11000 |
| Akaike Inf. Crit. | 228,963.90000 | 95,897.15000 | 190,556.20000 |
| *Note:* | + p<0.1; * p<0.05; ** p<0.01; *** p<0.001 | | |

** Other Race-Ethnicity:*
*American Indian or Alaska Native, Asian, Native Hawaiian, Native Hawaiian or Other Pacific Islander and Not Listed*

© 2023 Sangal RB et al. *JAMA Network Open.*

**eTable 6.** Regression Results Showing the Association Between UQJ Exposure and ED Care Outcomes

The first column shows the regression result for UQJ exposure on the likelihood of hallway placement, the second column shows the regression result for UQJ exposure on the likelihood of early ED departure (AMA, Eloped, LWBS), the third column shows the regression result for UQJ exposure on the probability of 72-hour revisit, and the fourth column shows the relationship between UQJ exposure and the likelihood of ED escalation. Coefficients presented are odds ratios where values below 1.0 represent a decrease in the odds, and values above 1.0 represent an increase in the odds.

**Association Between UQJs and ED Outcome Measures**

| | *Dependent variable:* | | | |
|---|---|---|---|---|
| | Hallway Placement | AMA / Eloped / LWBS | 72-Hr Revisit | ED Escalation |
| | OR (95%CI LB,UB) | OR (95%CI LB,UB) | OR (95%CI LB,UB) | OR (95%CI LB,UB) |
| | (1) | (2) | (3) | (4) |
| Passed over(s) by lower acuity patients | 1.005(0.995,1.016) | 1.029*(1.002,1.057) | 0.956(0.904,1.01) | 1.003(0.928,1.084) |
| Passed over(s) by same acuity patients | 1.01**(1.004,1.016) | 1.024**(1.006,1.042) | 1.013(0.975,1.052) | 0.938(0.867,1.015) |
| Received care ahead of higher acuity patients | 0.865***(0.827,0.904) | 0.907(0.805,1.021) | 0.984(0.789,1.229) | 0.916(0.648,1.295) |
| Received care ahead of same acuity patients | 1.09***(1.056,1.124) | 0.868**(0.782,0.963) | 0.974(0.841,1.129) | 1.063(0.925,1.221) |
| Age | 1(0.999,1) | 0.985***(0.982,0.989) | 1.005**(1.001,1.01) | 0.996+(0.992,1) |
| Arrival Method: Walk-In (ambulance as baseline) | 0.883***(0.857,0.909) | 1.164***(1.065,1.272) | 0.647***(0.551,0.761) | 0.636***(0.536,0.754) |
| High Utilizer | 1.174***(1.136,1.212) | 1.651***(1.493,1.825) | 12.114***(9.791,14.989) | 0.89(0.771,1.027) |
| Sex: Male (Female baseline) | 1.096***(1.07,1.122) | 1.229***(1.136,1.329) | 1.948***(1.643,2.309) | 1.066(0.968,1.173) |
| ESI3(ESI 2 as baseline) | 1.031*(1.003,1.059) | 1.298***(1.158,1.455) | 0.816*(0.679,0.981) | 0.457***(0.364,0.574) |
| ESI4(ESI 2 as baseline) | 1.372***(1.299,1.449) | 1.279***(1.102,1.484) | 0.865(0.689,1.087) | 0.223***(0.077,0.642) |
| Transfer In: Yes | 0.868***(0.823,0.915) | 0.419***(0.289,0.608) | 0.289***(0.132,0.632) | 1.054(0.914,1.216) |
| Race-Ethnicity: Black non-Hispanic (White non-Hispanic as baseline) | 0.992(0.969,1.015) | 0.887***(0.819,0.961) | 0.946(0.852,1.052) | 0.917(0.801,1.05) |
| Race-Ethnicity: Hispanic or Latino (White non-Hispanic as baseline) | 0.987(0.958,1.016) | 0.8***(0.714,0.896) | 0.403***(0.304,0.534) | 0.913(0.739,1.128) |
| Race-Ethnicity: Others (White non-Hispanic as baseline) | 1.022(0.968,1.078) | 0.67***(0.522,0.861) | 0.755(0.488,1.168) | 0.769(0.534,1.109) |
| Insurance: Medicaid (private insurance as baseline) | 1.04**(1.01,1.071) | 2.318***(1.952,2.752) | 2.485***(1.616,3.821) | 0.853(0.697,1.044) |
| Insurance: Medicare (private insurance as baseline) | 0.979(0.948,1.012) | 1.419***(1.203,1.673) | 1.806**(1.192,2.738) | 1.002(0.863,1.164) |
| Insurance: Other (private insurance as baseline) | 1.077***(1.032,1.124) | 1.718***(1.406,2.101) | 1.384(0.892,2.146) | 0.813(0.56,1.178) |
| Language: Other (English as baseline) | 0.956(0.893,1.023) | 0.58***(0.412,0.818) | 0.614(0.283,1.333) | 1.455*(1.023,2.067) |
| Language: Spanish (English as baseline) | 0.984(0.947,1.022) | 0.674***(0.566,0.802) | 1.475**(1.093,1.99) | 1.044(0.805,1.353) |
| ED Level Controls: | YES | YES | YES | YES |

© 2023 Sangal RB et al. *JAMA Network Open.*

| | | | | |
|---|---|---|---|---|
| Staffing Level Control | YES | YES | YES | YES |
| Patient mixture Control | YES | YES | YES | YES |
| Day of Week Control (factor) | YES | YES | YES | YES |
| Time of Day Control (hour, factor) | YES | YES | YES | YES |
| Year Control (year, factor) | YES | YES | YES | YES |
| Patient Residential Zipcode Control (factor) | YES | YES | YES | YES |
| Patient Chief Complaint Control (factor) | YES | YES | YES | YES |
| Constant | 0.041***(0.027,0.062) | 0.007***(0.002,0.02) | 0.002***(0,0.014) | 0.027***(0.004,0.19) |
| Observations | 314,392 | 314,392 | 213,668 | 93,850 |
| Log Likelihood | -165,933.100 | -30,912.620 | -9,773.983 | -11,127.070 |
| Akaike Inf. Crit. | 332,116.200 | 62,079.230 | 19,805.970 | 22,510.140 |

*Note:* + p<0.1; * p<0.05; ** p<0.01; *** p<0.001

** Other Race-Ethnicity:*
*American Indian or Alaska Native, Asian, Native Hawaiian, Native Hawaiian or Other Pacific Islander and Not Listed*

**eFigure 3.** Summary of Regression Results for Secondary Analysis on a Subset of High Acuity Patient Encounters Only.
Left panel: occurrence of being passed over by lower acuity patients for high acuity encounters.
Right panel: occurrence of being passed over by same acuity patients for high acuity encounters.
We did not examine receiving care ahead of higher acuity patients and receiving care ahead of same acuity patients in this subset because the former is not defined for ESI-1 visits, and the latter is a very rare event for ESI-1 visits.

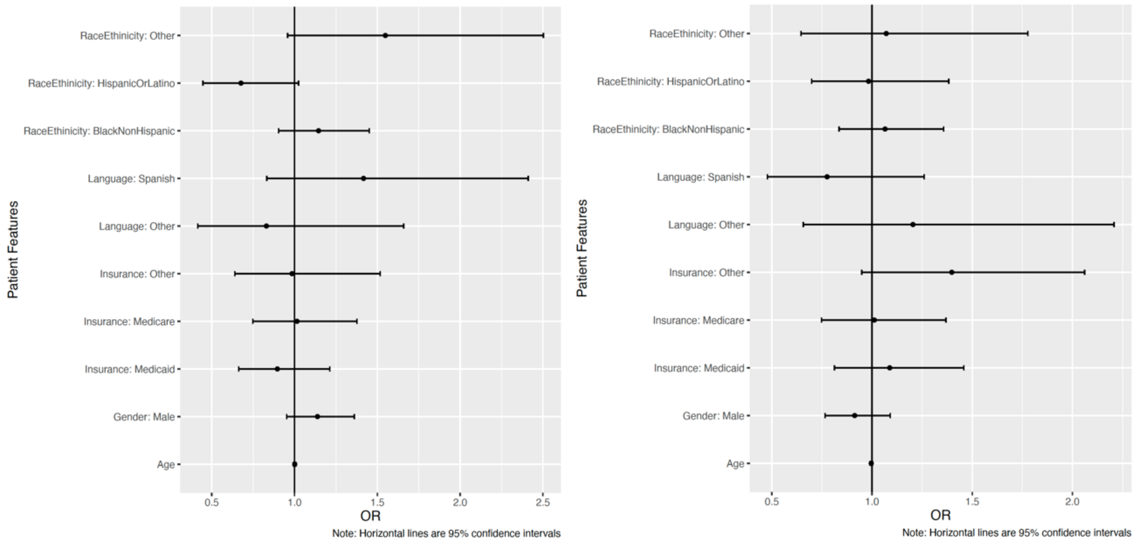


**eTable 7.** Table of Regression Results for Secondary Analysis on a Subset of High Acuity Patient Encounters Only.

© 2023 Sangal RB et al. *JAMA Network Open.*

The first column shows the regression coefficients for being passed over by lower acuity patients and the second column shows the regression coefficients for being passed over by same acuity patients.

**Social Risk Factors for Being Passed Over**
**(Subset Analysis on Higher Acuity Encounters)**

| | *Dependent variable:* | |
|---|---|---|
| | *Passed over by lower acuity patients* OR (95%CI LB,UB) (1) | *Passed over by lower acuity patients* OR (95%CI LB,UB) (2) |
| Age | 1.001 (0.994, 1.008) | 0.996 (0.990, 1.003) |
| Arrival Method: Walk-In (ambulance as baseline) | 1.130 (0.878, 1.455) | 1.203 (0.952, 1.520) |
| High Utilizer | 1.020 (0.780, 1.334) | 0.918 (0.697, 1.209) |
| Sex: Male (Female baseline) | 1.138 (0.952, 1.361) | 0.914 (0.765, 1.090) |
| ESI2 (ESI 1 as baseline) | 0.630*** (0.496, 0.798) | 21.753*** (10.089, 46.901) |
| Transfer In: Yes | 1.104 (0.846, 1.441) | 0.974 (0.736, 1.290) |
| Race-Ethnicity: Black non-Hispanic (White non-Hispanic as baseline) | 1.145 (0.903, 1.451) | 1.065 (0.836, 1.357) |
| Race-Ethnicity: Hispanic or Latino (White non-Hispanic as baseline) | 0.676+ (0.447, 1.024) | 0.983 (0.698, 1.383) |
| Race-Ethnicity: Others (White non-Hispanic as baseline) | 1.548+ (0.958, 2.503) | 1.071 (0.646, 1.777) |
| Insurance: Medicaid (private insurance as baseline) | 0.896 (0.662, 1.213) | 1.088 (0.812, 1.458) |
| Insurance: Medicare (private insurance as baseline) | 1.015 (0.748, 1.376) | 1.011 (0.748, 1.368) |
| Insurance: Other (private insurance as baseline) | 0.985 (0.639, 1.518) | 1.398+ (0.948, 2.061) |
| Language: Other (English as baseline) | 0.830 (0.415, 1.659) | 1.204 (0.657, 2.208) |
| Language: Spanish (English as baseline) | 1.417 (0.832, 2.411) | 0.776 (0.478, 1.261) |
| ED Level Controls: | YES | YES |
| Staffing Level Control | YES | YES |
| Patient mixture Control | YES | YES |
| Day of Week Control (factor) | YES | YES |
| Time of Day Control (hour, factor) | YES | YES |
| Year Control (year, factor) | YES | YES |
| Patient Residential Zipcode Control (factor) | YES | YES |
| Patient Chief Complaint Control (factor) | YES | YES |

© 2023 Sangal RB et al. *JAMA Network Open.*

| | | |
|---|---|---|
| Constant | 0.039* (0.002, 0.654) | 0.060+ (0.002, 1.674) |
| Observations | 10,634 | 10,634 |
| Log Likelihood | -2,103.787 | -2,078.351 |
| Akaike Inf. Crit. | 4,443.575 | 4,392.701 |
| *Note:* | | + p<0.1; * p<0.05; ** p<0.01; *** p<0.001 |

** Other Race-Ethnicity:*
*American Indian or Alaska Native, Asian, Native Hawaiian, Native Hawaiian or Other Pacific Islander and Not Listed*

© 2023 Sangal RB et al. *JAMA Network Open.*
