# Supplementary material for: Sociodemographic Disparities in Queue Jumping for Emergency Department Care
Source: JAMA Netw Open. 2023 Jul 28;6(7):e2326338. doi: 10.1001/jamanetworkopen.2023.26338 (PMC10383013; doi:10.1001/jamanetworkopen.2023.26338)
Supplement: Supplement 2. — Data Sharing Statement [file jamanetwopen-e2326338-s002.pdf]

Data Sharing Statement

Sangal. Sociodemographic Disparities in Queue Jumping for Emergency Department Care. *JAMA Netw Open*. Published July 28, 2023. doi:10.1001/jamanetworkopen.2023.26338

Data
Data available: No

Additional Information
Explanation for why data not available: Not approved for data sharing outside institution
